# How to convert urban energy balance into contributions to urban excess temperatures?

**DOI:** 10.1016/j.mex.2018.12.015

**Published:** 2018-12-27

**Authors:** Daniel Hertel, Uwe Schlink

**Affiliations:** Department of Urban and Environmental Sociology, Helmholtz Centre for Environmental Research – UFZ, Permoserstraße 15, D-04318, Leipzig, Germany

**Keywords:** Urban heat island decomposition, Urban heat island, ENVI-met, Urban heat attribution

## Abstract

A new approach partitioning the urban heat island intensity (ΔT) into its contributing processes is developed for the neighbourhood scale. The method transforms individual terms of the energy balance (radiation, evapotranspiration, heat storage, and convection) into partitions of temperature and is exemplified using the output of a micrometeorological model.

• The temperature contribution is determined by climate sensitivity and a gain function depending on the energy redistribution factor.
• The method is exemplified for the output of ENVI-met.
• The software implementing the method is written in R language, a free language enabling statistical computations.

## Graphical abstract

**Figure:**
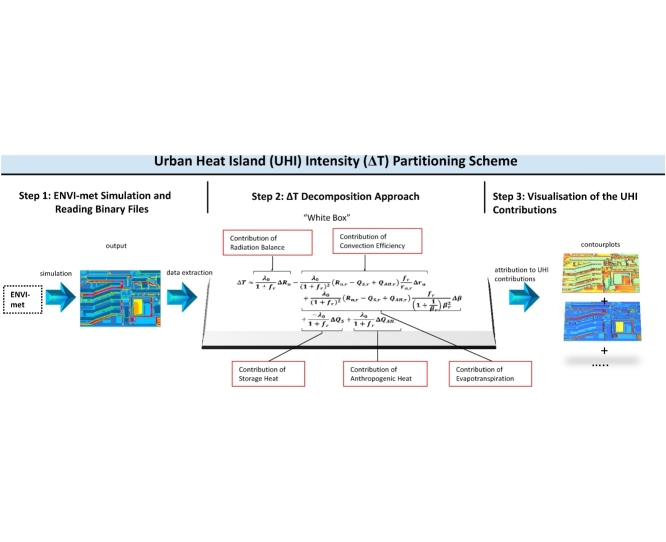

**Specifications Table**

**Table:** 

Subject area	*Environmental Science*
More specific subject area	*Urban thermal conditions*
Method name	*Urban heat island decomposition*
Name and reference of original method	*The method was inspired by a previous paper of Zhao, L., X. Lee, R. B. Smith and K. Oleson (2014). "Strong contributions of local background climate to urban heat islands." Nature 511(7508): 216-219.*
Resource availability	*The software implementing the Urban Heat Island decomposition has been written in R language (*www.r-project.com) and the relevant R scripts are reported in this manuscript to allow the reproducibility of the results. ENVI-met is available from www.envi-met.com.

## Method details

Attribution techniques are defined as methods “…evaluating the relative contributions of multiple causal factors to a change or event…” [1]. Typically they are used for decomposing climate change into its causes and determining the magnitude of the anthropogenic contribution. Such techniques can be utilised for different changes or events whereby one of them is the Urban Heat Island (UHI) effect that represents a temperature difference between urban and rural areas. The identification of dominant factors to UHI can improve the understanding of their dynamics and formation.

Often, UHI attribution focuses on the city scale [2,3] or only on the respective contributions of the surface energy balance (SEB) terms. The specific underlying processes responsible for that and their caused temperature change are not considered (e.g. [4]). At smaller scales, like building levels, the urban geometry becomes very complex, which confines such investigations to single objects or street canyons with predefined orientations. The focus on the identification of dominant processes again lacks of a statement to the caused temperature change (e.g. [5]).

**Fig. 1:**
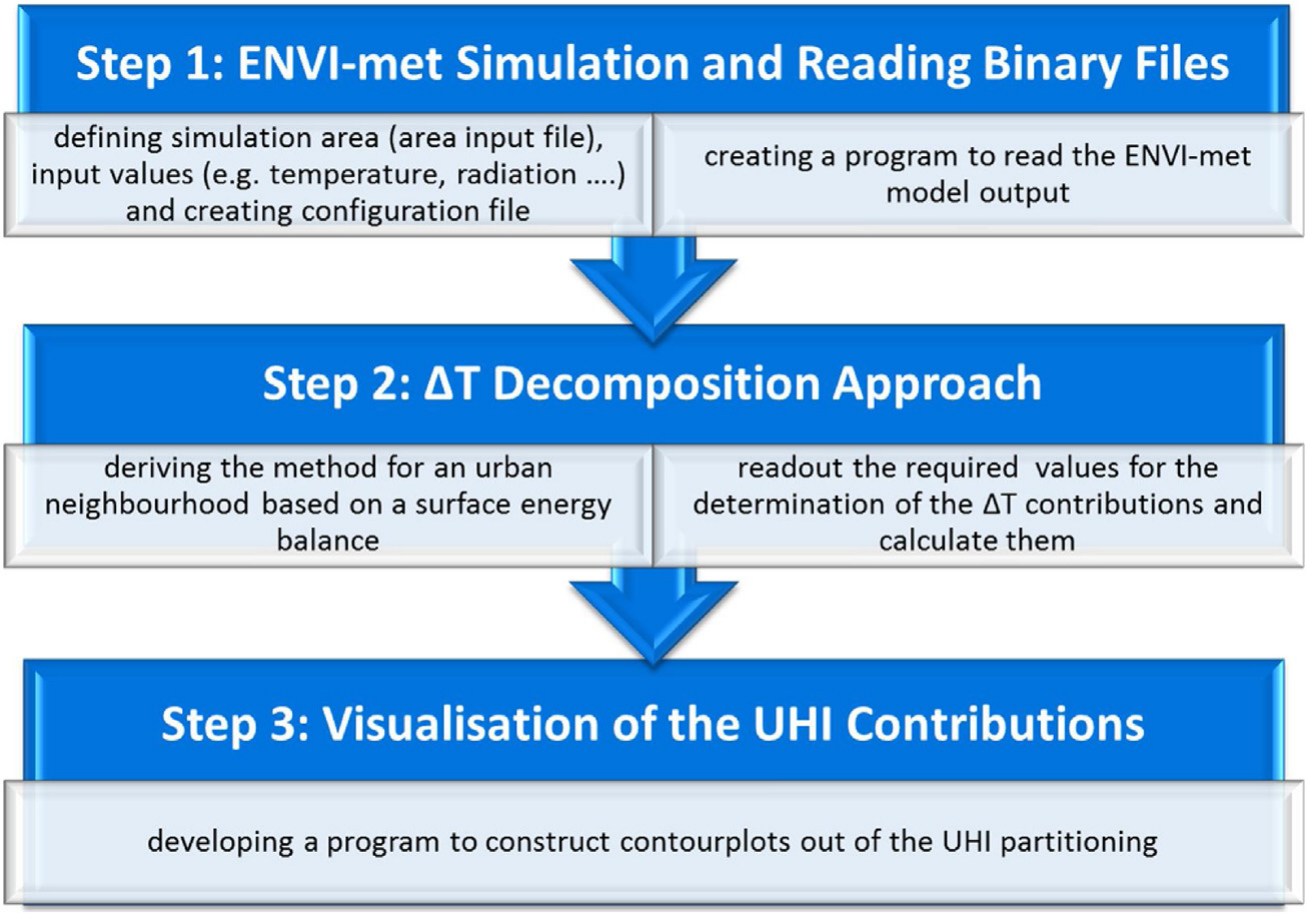
Methodological framework.

In the present publication, we deduce a method for decomposing the Urban Heat Island intensity (ΔT) at the neighbourhood scale. The procedure is guided by a previously published approach [6]. In addition we share and describe an R [9] code especially developed for the decomposition scheme. The code is part of the work flow shown in Fig. 1 and involves three main steps that are explained in the subparagraphs below.

## Step 1: ENVI-met simulation and reading binary files

The micrometeorological model ENVI-met (version 3.1, [7]) was applied to simulate all necessary meteorological field variables in the neighbourhood of interest. The presented code can be easily extended and adapted to the needs of the user.

Although the formation of an UHI effect requires low wind speeds, it has to be noted that the initial wind speed should be not smaller than 3 m/s. Otherwise the model can crash because of emerging numerical instabilities. The specific humidity at 2500 m (top of model atmosphere) was approximately derived from radio soundings. The choice of the lateral boundary conditions depends on the surroundings of the study area. A comparison with measurements showed that the combination of open (for temperature and specific humidity) – forced (for turbulent kinetic energy) boundary conditions was best for our purposes. Anyway, it is highly recommendable to test different combinations in order to achieve the most accurate simulation results.

Further details about model specifications and configurations for a specific case study are presented in [8] since these vary according to the local characteristics.

### Reading ENVI-met output

The ENVI-met model output is binary and stored within the respective “*.EDT” files. Since the corresponding “*.EDI” files provide information about the data structure, we wrote an R-program extracting all meteorological fields. For any variable, the fields (matrices) of each height level (z = 0…29) are arranged one below the other starting with z = 0. To handle this structure, our code is divided into three parts: First, the binary format needs to be read into R and converted into a proper format. Second, we loop until we reach the variable of interest (loop index can be derived from “*.EDI” file by counting the position) and then, in a second loop, we select the height level of interest. Third, the resulting matrix is stored as a text file.

### R program definitions

**Fig. 2:**
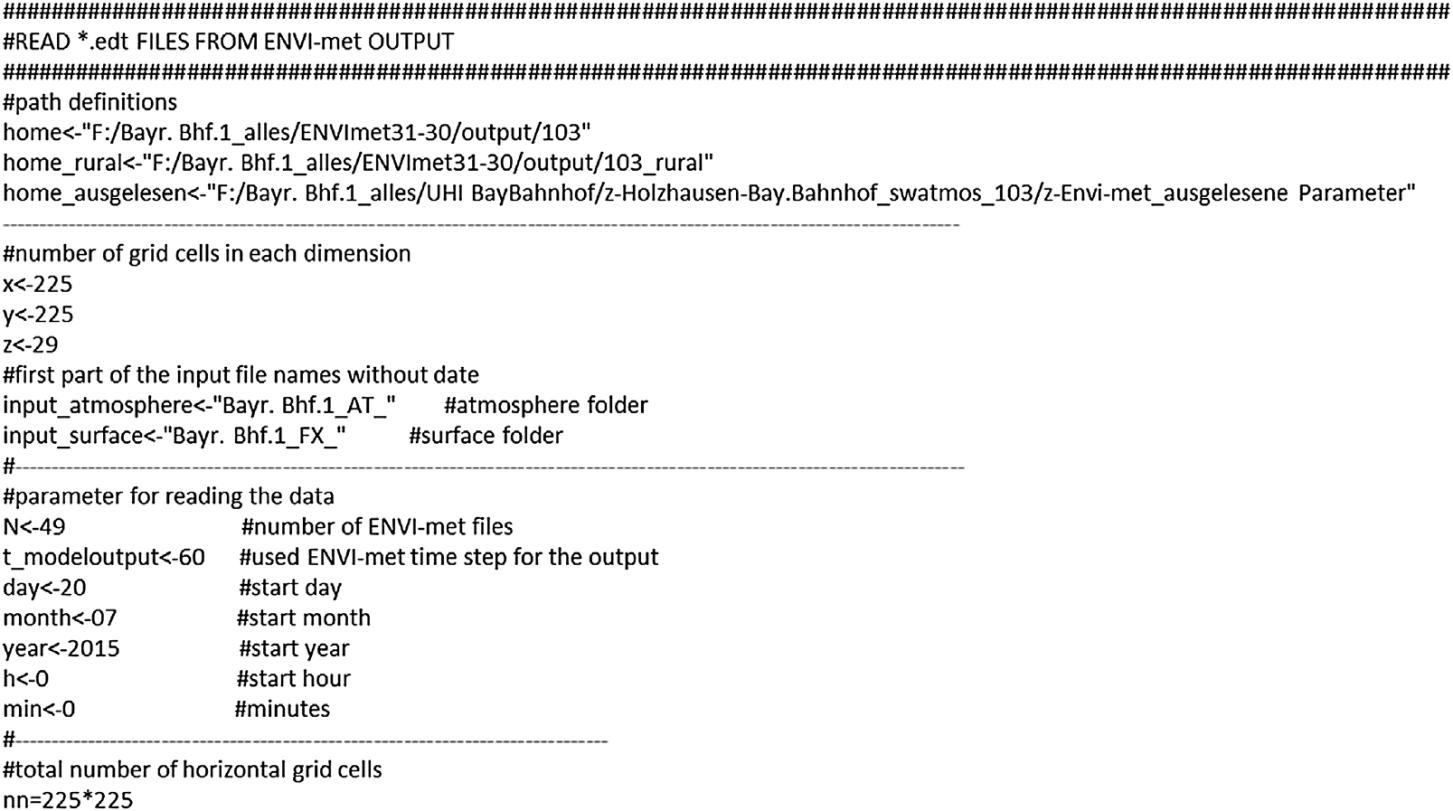
Prespecified program variables to readout ENVI-met output.

The subsequent code exemplifies this procedure for the sky view factor. The first lines (Fig. 2) define the number of grid cells, paths to the ENVI-met output folders and the location where the converted files should be stored as well as further parameters for reading the data.

### Extracting required variables

**Fig. 3:**
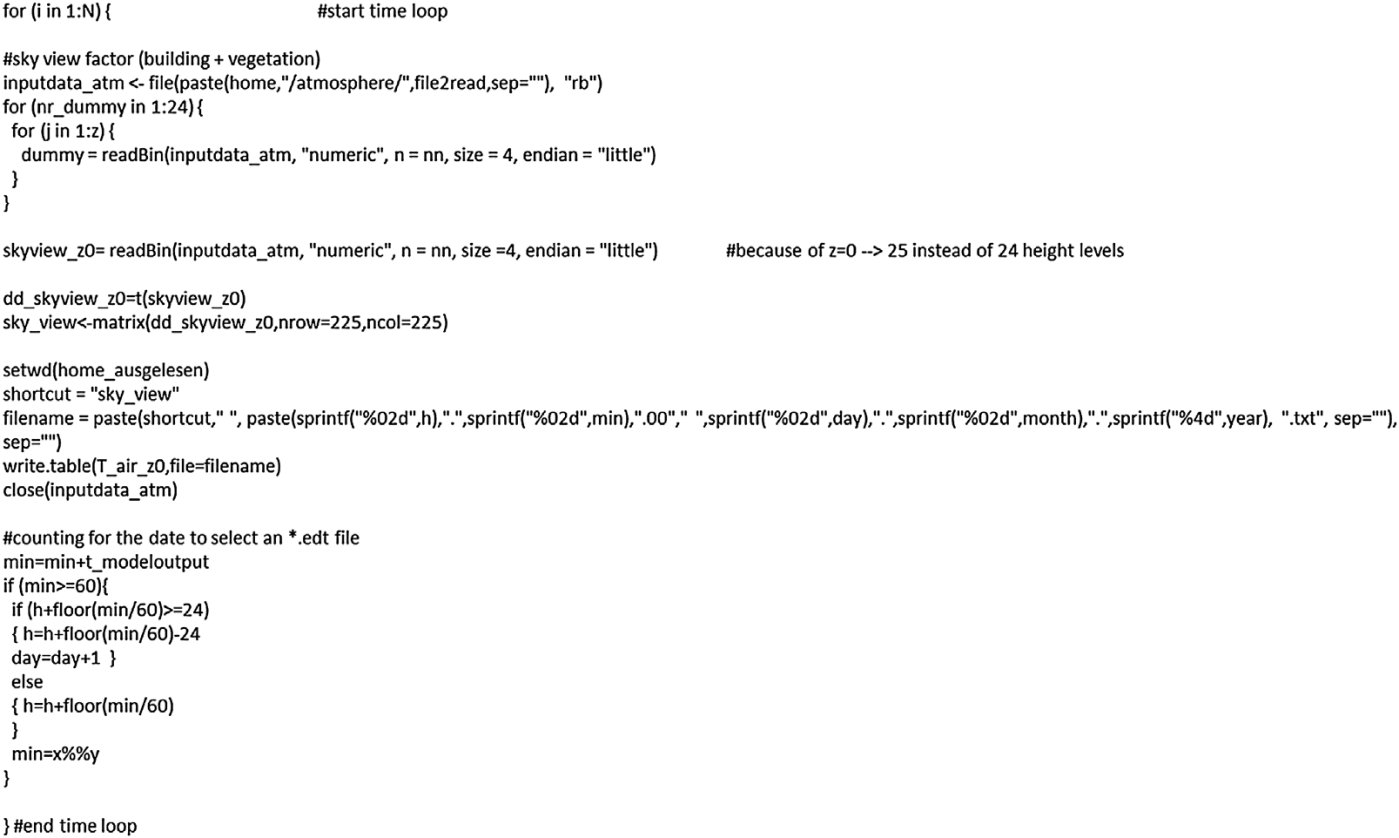
Converting variables of interest from binary to numeric matrix format.

The following lines (Fig. 3) convert the binary files into matrices. The first for-loop refers to the number of ENVI-met output files. The second one selects the sky view factor (here position 25) and the third one overwrites all previous height levels for the variables that are not used. In result, we receive the sky view factor at z = 0 (that is 0.5 m above surface) in a vector format. For later calculations, it is converted into a matrix.

This simple code can be modified and extended according to the variables needed or written as a function with arguments (e.g. “nrow”, “ncol”; area size).

## Step 2: ΔT decomposition approach

### Theory

Pursuant to the formulation of [6], we interpret the decomposition approach for an urban neighbourhood. An urban neighbourhood results from a land use change compared to rural structures. This change is considered as a perturbation to the rural state. In this way, ΔT represents the UHI intensity in the respective neighbourhood. In order to quantify different heat flux contributions that are caused by the perturbation, we formulate the energy balance of an arbitrary surface in terms of radiation balance, turbulent heat fluxes, heat storage, and the anthropogenic induced heat flux [8].

Together with both sensible heat flux

(1) $$Q_{H}=\rho c_{p}\frac{T-T_{a}}{r_{a}}$$

and latent heat flux

(2) $$Q_{LE}=\frac{Q_{H}}{\beta }$$

this yields the surface energy balance (without advection)

(3) $$\underset{R_{n}}{\underbrace{\left(1-a\right)S_{↓}+L_{↓}-\varepsilon \sigma T^{4}}}+Q_{AH}=\underset{Q_{H}+Q_{LE}}{\underbrace{\left(1+\frac{1}{\beta }\right)\frac{\rho c_{p}}{r_{a}}\left(T-T_{a}\right)}+Q_{S}}$$

where $R_{n}$ is the net radiation balance, $S_{↓}$ the incoming short-wave radiation, $L_{↓}$ the incoming long-wave radiation, $Q_{S}$ the storage heat flux, a the albedo, ε the surface emissivity, σ the Stefan – Boltzmann constant, ρ the air density, $c_{p}$ the specific heat of air at constant pressure (1013.25 hPa), $T_{a}$ the air temperature at a reference height, $r_{a}$ the aerodynamic resistance to heat diffusion and $\beta =Q_{H}/Q_{LE}$ the Bowen ratio. Positive fluxes are directed away from the surface, negative ones are directed towards the surface.

For two sites with different surface characteristics, we can assume that at a certain height (reference height) the influence of the surface on the air temperature vanishes. In this case $T_{a}$ is equal for both sites and we linearise the emitted long-wave radiation in Eq. (3) at this point. After rearranging and factorising of $T-T_{a}$ we obtain

(4) $$R_{n}-Q_{S}+Q_{AH}=\left[\left(1+\frac{1}{\beta }\right)\frac{\rho c_{p}}{r_{a}}+4\varepsilon \sigma T_{a}^{3}\right]\left(T-T_{a}\right).$$

Isolating $T-T_{a}$ finally yields

(5) $$T-T_{a}=\frac{\lambda_{0}}{1+f}\left(R_{n}-Q_{S}+Q_{AH}\right),$$

with

$$f=\frac{\lambda_{0}\rho c_{p}}{r_{a}}\left(1+\frac{1}{\beta }\right),\;\lambda_{0}=\frac{1}{4\varepsilon \sigma T_{a}^{3}}$$

**Fig. 4:**
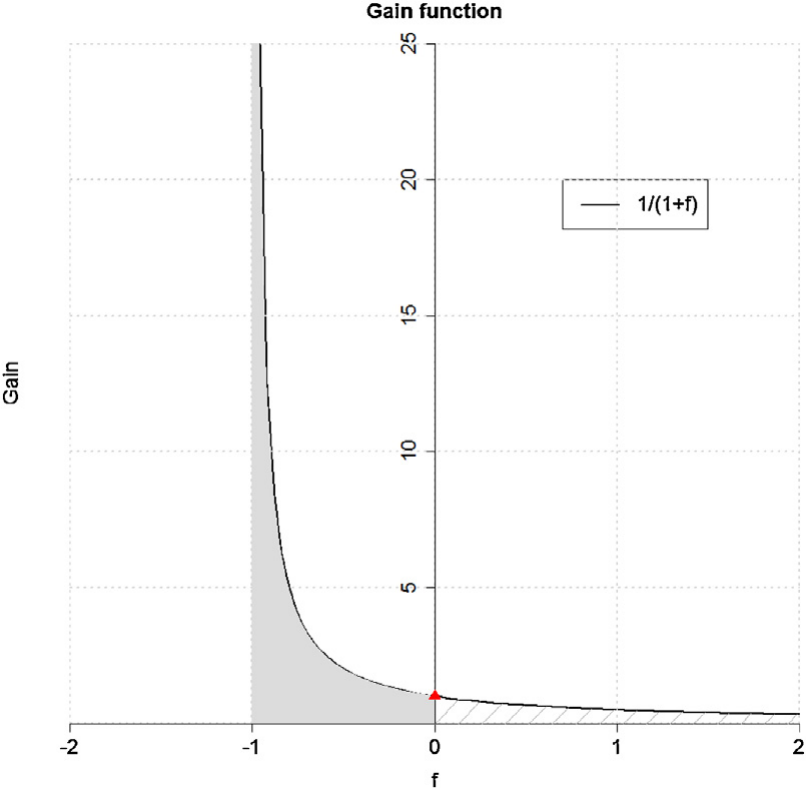
Gain as a function of the energy redistribution factor f. The dark greyish contour denotes amplification and the grey hatched region indicates damping. At the red triangle, the gain function is 1 indicating the transition between both cases.

Eq. (5) translates the surface energy balance into temperature differences [8]. If we consider a particular state as reference (“rural state”) then the other one represents a perturbation (”urban state”) caused by energy redistribution (right hand side of Eq. (5)). f is the fraction of energy redistribution associated with turbulent processes, which can either damp or amplify the reaction at the reference site to the perturbation. For that reason, $G(f)=1/\left(1+f\right)$ indicates the gain resulting from such turbulent redistribution processes. The gain function G (Fig. 4) tends to amplify (G>1) for $-1<f<0\;\left(\beta <0\right)$ and to damp (0<G<1) for f>0. A negative gain is physically impossible and f has to be greater than −1. $\lambda_{0}$ translates an energy flux to a temperature response (in Eq. (5)) and is called climate sensitivity.

In an urban environment, we consider the difference between urban and rural state. Applying this to Eq. (5) yields

(6) $$T_{u}{-T}_{r}=\frac{\lambda_{0}}{1+f_{u}}\left(R_{n,u}-Q_{S,u}+Q_{AH,u}\right)-\frac{\lambda_{0}}{1+f_{r}}\left(R_{n,r}-Q_{S,r}+Q_{AH,r}\right).$$

The temperature of the urban state $T_{u}$ can be replaced by the temperature of the rural state plus a perturbation caused by the urban structures ($T_{r}+\text{Δ}T$). Analogous replacements apply for $f_{u}$, $R_{n,u}$, $Q_{S,u}$ and $Q_{AH,u}$. Δ indicates small perturbations. Inserting these replacements into Eq. (6) and calculating the derivatives results in the UHI intensity (ΔT) of an urban neighbourhood

(7) $$\text{Δ}T=\frac{\lambda_{0}}{1+f_{r}}\left(R_{n,r}+\text{Δ}R_{n}-Q_{S,r}-\text{Δ}Q_{S}+Q_{AH,r}+\text{Δ}Q_{AH}\right)-\frac{\lambda_{0}}{1+f_{r}}\left(R_{n,r}-Q_{S,r}+Q_{AH,r}\right)-\;\frac{\lambda_{0}}{{\left(1+f_{r}\right)}^{2}}\left(R_{n,r}+\text{Δ}R_{n}-Q_{S,r}-\text{Δ}Q_{S}+Q_{AH,r}+\text{Δ}Q_{AH}\right)\text{Δ}f.$$

Neglecting higher order terms $O\left({\text{Δ}}^{2}\right)$, it follows

(8) $$\text{Δ}T\approx \frac{\lambda_{0}}{1+f_{r}}\text{Δ}R_{n}-\frac{\lambda_{0}}{{\left(1+f_{r}\right)}^{2}}\left(R_{n,r}-Q_{S,r}+Q_{AH,r}\right)\text{Δ}f-\frac{\lambda_{0}}{1+f_{r}}\text{Δ}Q_{S}+\frac{\lambda_{0}}{1+f_{r}}\text{Δ}Q_{AH}.$$

Evaluating $\text{Δ}f\left(r_{a},\beta \right)$ we find for $\text{Δ}T\left(x,t\right)$

(9) $$\text{Δ}T\approx \frac{\lambda_{0}}{1+f_{r}}\text{Δ}R_{n}-\frac{\lambda_{0}}{{\left(1+f_{r}\right)}^{2}}\left(R_{n,r}-Q_{S,r}+Q_{AH,r}\right)\frac{f_{r}}{r_{a,r}}\text{Δ}r_{a}+\frac{\lambda_{0}}{{\left(1+f_{r}\right)}^{2}}\left(R_{n,r}-Q_{S,r}+Q_{AH,r}\right)\frac{f_{r}}{\left(1+\frac{1}{\beta_{r}}\right)\beta_{r}^{2}}\text{Δ}\beta +\frac{{-\lambda }_{0}}{1+f_{r}}\text{Δ}Q_{S}+\frac{\lambda_{0}}{1+f_{r}}\text{Δ}Q_{AH.\;}$$

Eq. (9) is an approximation of the UHI intensity. All parameters on the right hand side of Eq. (9) that are associated with Δ characterising changes in the energy balance between a rural area and an urban neighbourhood. All other quantities, including $\lambda_{0}/\left(1+f\right)$, convert the changes in the respective energy term into a temperature change whose sum result in the UHI intensity for each model cell and time step in the quarter.

### R program definitions

**Fig. 5:**
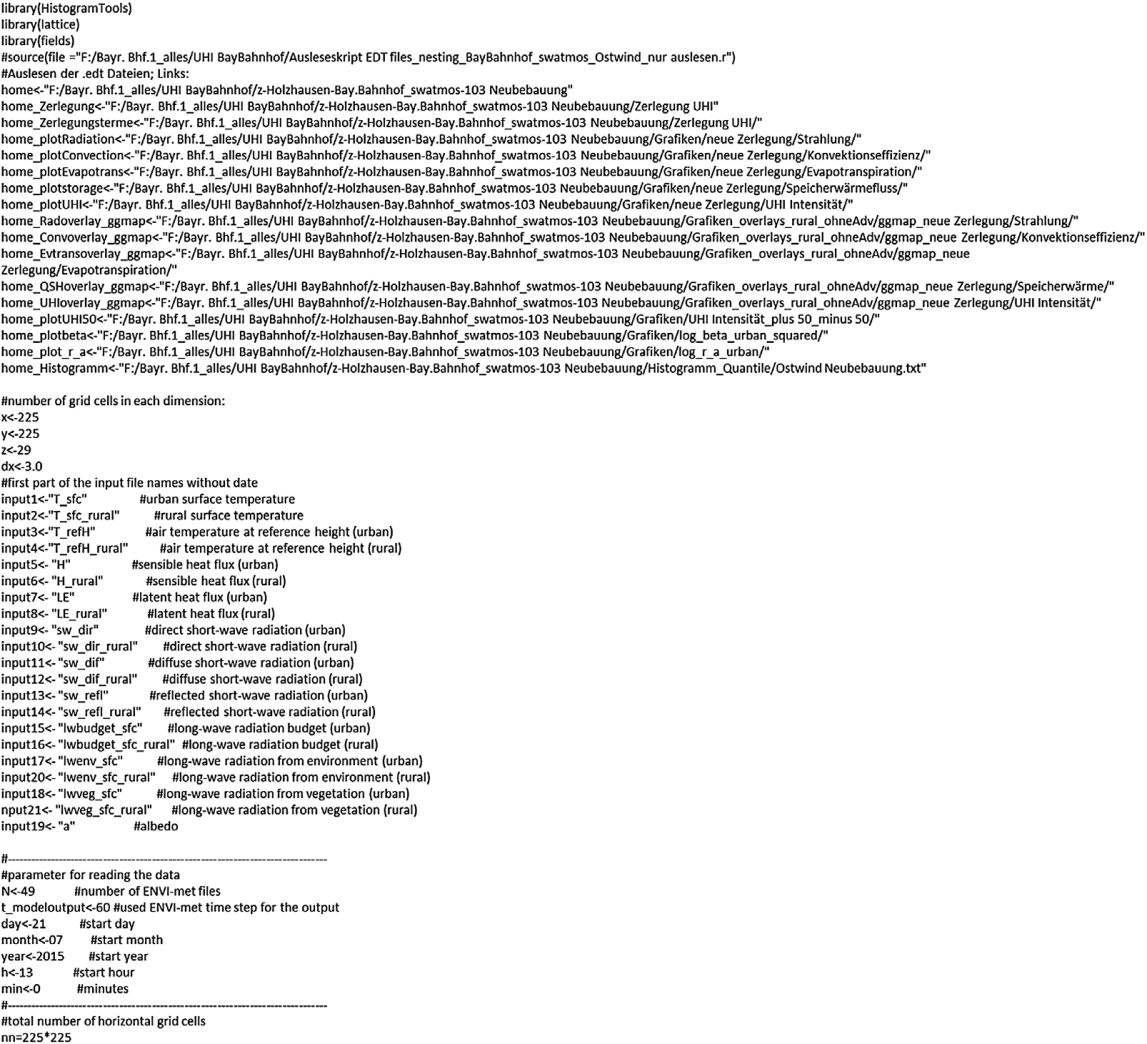
Prespecified program variables to decompose ΔT.

The first lines of this code (Fig. 5) define needed libraries, information about the locations where the results should be stored as well as the folder where the extracted ENVI-met variables are located.

### Start of time loop and reading of the data

**Fig. 6:**
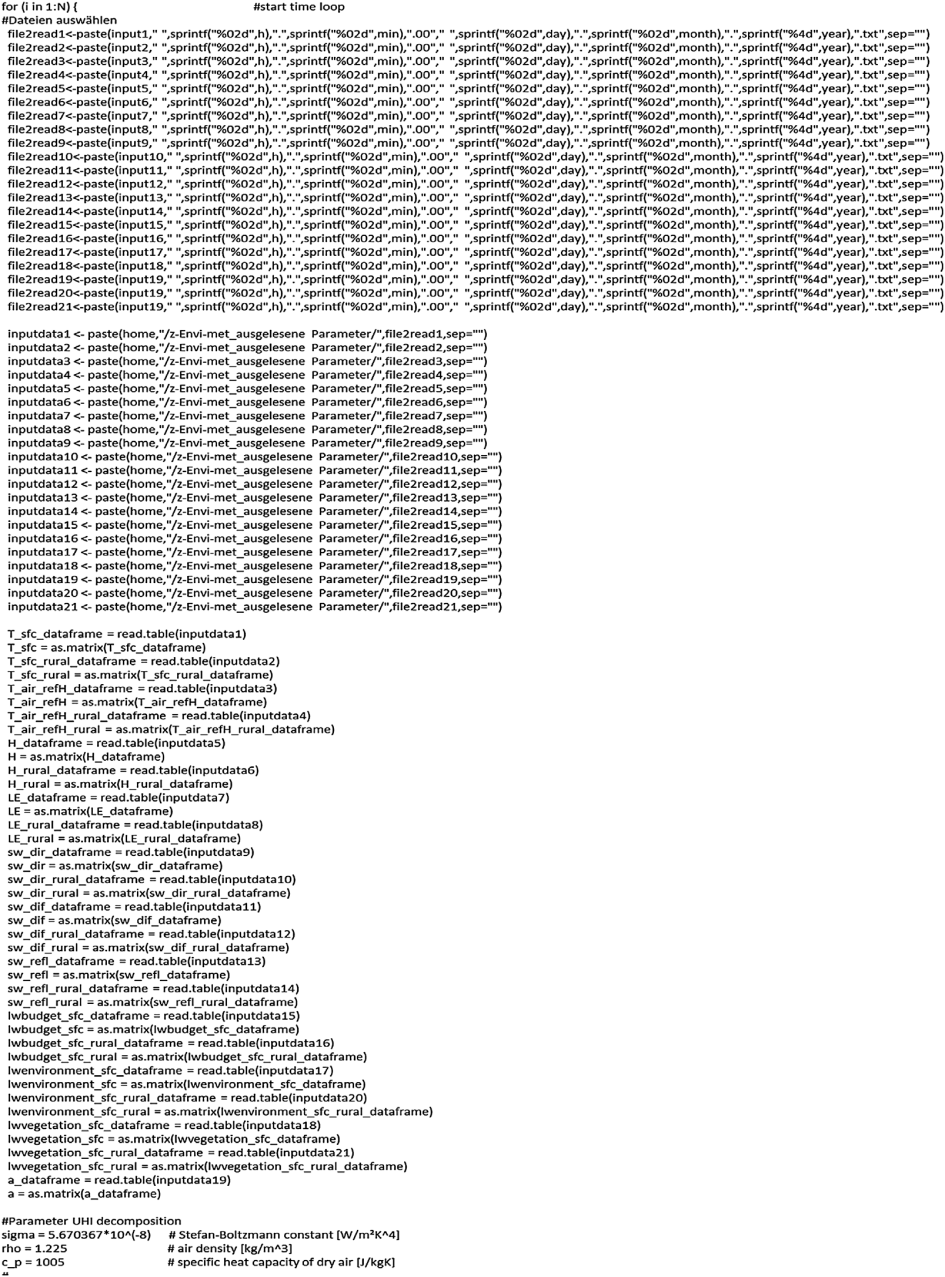
Begin of the time-loop over all output files to achieve necessary input values for ΔT decomposition.

Next (Fig. 6), the time loop over all available files starts (loop counter equal to the number of ENVI-met output files). After constructing the complete path names of all input variables they have to be converted into a matrix since the chosen visualisation function needs this kind of class type. Finally, to complete the definition and data reading part, some parameters for ΔT-decomposition are defined.

### ΔT decomposition: radiation

**Fig. 7:**
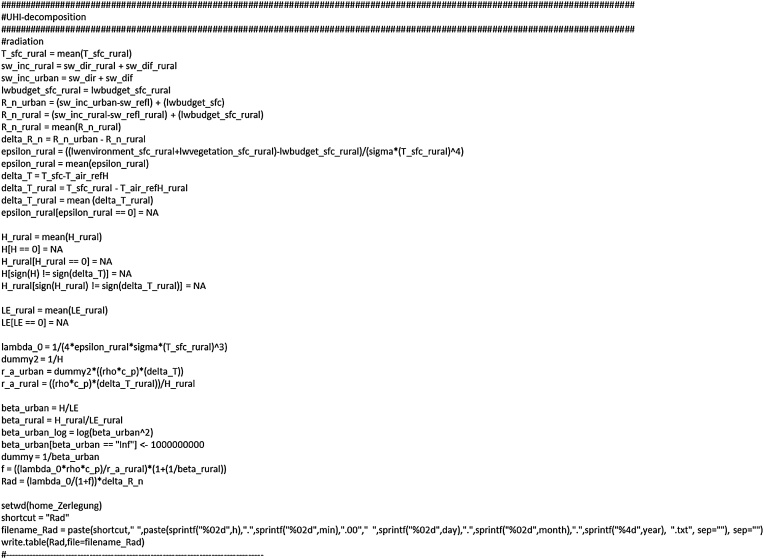
Calculating and attributing ΔT caused by changes in the radiation balance.

After these leading information, the first term in Eq. (9), characterising the change in radiation balance between the urban and rural state, is calculated (Fig. 7). As the surface emissivity is not available from the ENVI-met output we used the long-wave radiation budget (“lwbudget_sfc_rural”) and the incoming long-wave radiation (“lwenvironment_sfc_rural + lwvegetation_sfc_rural”) to derive this value for the rural state. The sensible (“H” (urban), “H_rural” (rural)) and latent heat fluxes (“LE” (urban), “LE_rural” (rural)) as well as the Bowen ratios (“beta_urban” and “beta_rural”) have to be filtered to avoid critical cases (further discussed in [8]) within the calculation.

### ΔT decomposition: convection and evapotranspiration

**Fig. 8:**
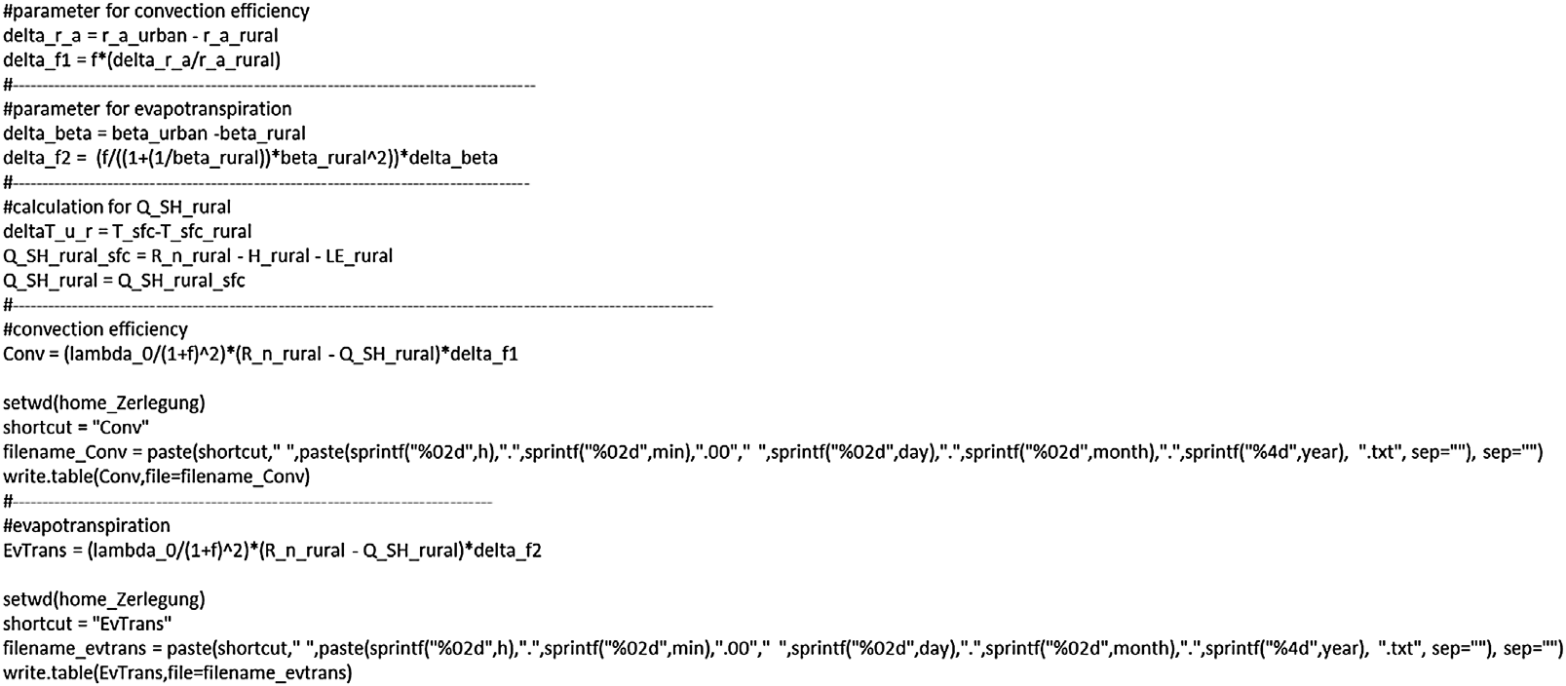
Calculating and attributing ΔT’s caused by changes in the convection efficiency and evapotranspiration.

The second (change in convection efficiency) and third (change in evapotranspiration) terms in Eq. (9) are determined by the following lines (Fig. 8)

### ΔT decomposition: Heat storage and total ΔT

**Fig. 9:**
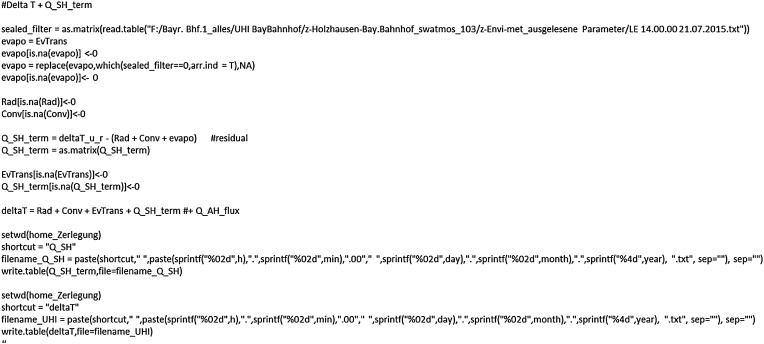
Calculating and attributing ΔT caused by changes in the heat storage and determining total ΔT.

Here, the anthropogenic heat flux ($Q_{AH}$) is neglected but can easily be implemented in the code. The effect of the change in heat storage (“Q_SH_term”) is calculated as a residual so that, together with the other 3 terms, the UHI intensity (“DeltaT”) for a neighbourhood is formed (Fig. 9). The variable “sealed_filter” and the subsequent 4 lines replace all sealed surfaces for the evapotranspiration term with zero values since these are impervious to water.

## Step 3: visualisation of ΔT contributions

**Fig. 10:**
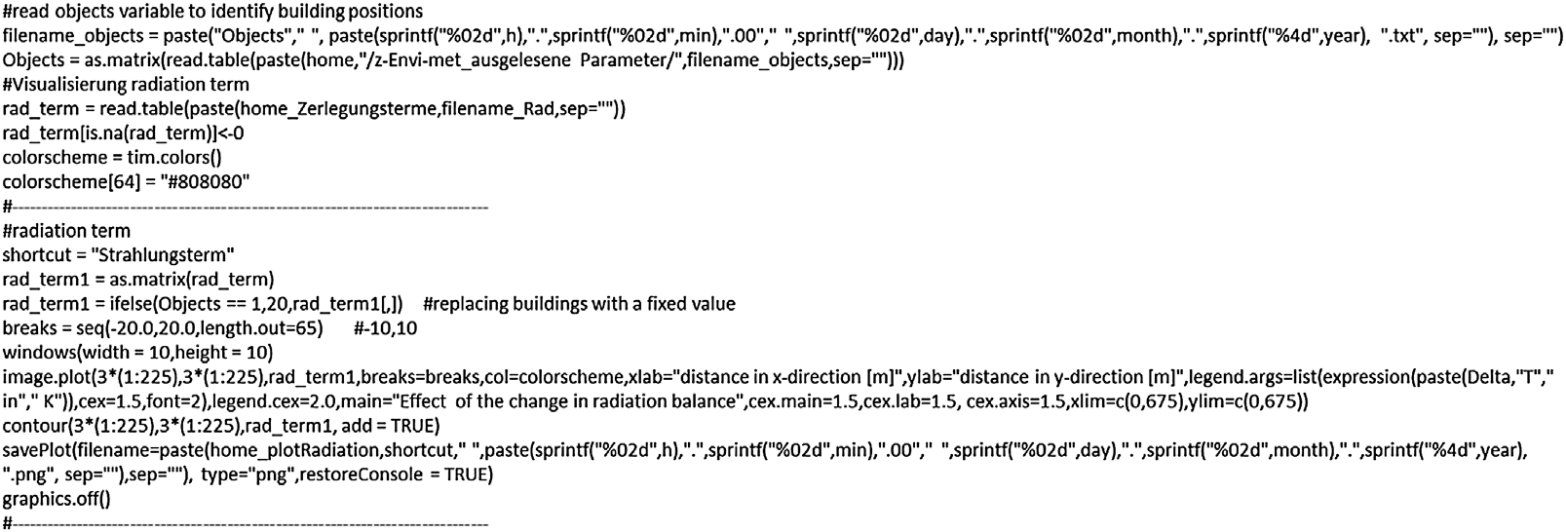
Visualisation of ΔT and its respective contributions with contour plots.

Using the program code described in step 1, all variables required for Eq. (9) are extracted. In step 2 the ΔT contributions are calculated. In step 3, applying the “image.plot” function, we exemplify the spatial visualisation for the contribution of the radiative energy (Fig. 10). This can be extended for the other terms of the decomposition procedure or further analysis.
